# Total Knee Replacement in the Obese Patient: Comparing Computer Assisted and Conventional Technique

**DOI:** 10.1155/2014/272838

**Published:** 2014-01-09

**Authors:** Yogeesh D. Kamat, Kamran M. Aurakzai, Ajeya R. Adhikari

**Affiliations:** Elective Orthopaedic Centre, Epsom, Surrey KT18 7EG, UK

## Abstract

*Purpose*. Obesity is being considered a “global epidemic.” Surgical procedures are rendered more difficult in obese patients. We aimed to see whether any benefits were evident with use of computer navigation during total knee replacement in these cases. *Methods*. A retrospective analysis of 287 TKR performed by a single surgeon was undertaken, including 133 TKR undertaken with computer navigation and 154 using standard instrumentation. Each group was further divided into subgroups depending on whether the patients were obese or nonobese. *Results*. We found that TKR in obese patients took longer with standard instruments, but not with computer navigation. A chronological analysis revealed that the surgeon progressively got quicker using computer navigation to the point that there was no difference in time with either of the operative techniques in obese patients. The mid-term clinical outcomes at five years were similar. Computer navigated TKR were more consistently accurately aligned. *Conclusions*. In obese patients, a dual advantage is provided by computer navigation: better alignment and no time penalty.

## 1. Introduction

Obesity is a growing problem in the modern world. The United Kingdom in the last decade has seen the worst demographic obesity trends in Europe [1]. The World Health Organisation (WHO) classifies body mass index (BMI) less than 25 kg/m^2^ as normal. BMI between 25 and 30 is graded as “overweight.” BMI greater than 30 is defined as “obese” and that greater than 40 called is “morbidly obese” [2]. Being obese is known to result in a higher risk for a number of chronic diseases [3].

Surgery in obese patients carries an increased risk of complications [4]. The probability of technical difficulties during total knee replacement (TKR) is hence also greater. In the past, there have been attempts to deny joint replacements to obese patients or to delay the operation in the hope that the patient would first lose weight [5]. Such action was subsequently criticized, as studies did not demonstrate a difference in clinical outcome of joint replacement in the obese [6, 7].

The use of computer navigation in TKR results in more consistently accurate alignment of the prosthetic components [8, 9]. This aid, however, comes with a time penalty [10]. Some early benefits of computer assisted TKR have been highlighted, for example, reduction in blood loss and systemic emboli [11, 12]. However, despite better alignment, computer navigation has not demonstrated better clinical outcomes as compared to TKR undertaken with standard instruments [13–15].

We aimed to compare the surgical times of TKR undertaken with and without the assistance of computer navigation, in obese and nonobese patients by a single surgeon in an arthroplasty unit. We also retrospectively analysed the early to mid-term clinical outcomes of these TKR.

## 2. Materials and Methods

A retrospective analysis of 287 TKR performed using the TC Plus SB implants (Plus Orthopaedics) by the senior author at the Elective Orthopedic Centre between 2006 and 2008 was performed. Of these, 133 TKR were undertaken using the computer navigation system. 154 TKR were performed by the conventional method (standard instruments). We have limited instrumentation sets to undertake computer navigation and can use these for about half the caseload of TKR. Patients are allocated to standard instruments or computer navigation by the scheduling clerical staff, purely on availability of instruments. Though our sample was not randomised, the surgical team did not have any control over the allocation of patients to technique of operation.

All patients were part of an arthroplasty outcome programme within this unit. Preoperative demographic parameters and Oxford Knee Scores (OKS) are recorded for all patients. Following surgery, the patients receive a postal questionnaire comprising the OKS every year. At three years postop they were called for a clinicoradiological followup visit when International Knee Society Scores (IKSS) and X-rays were undertaken. Radiographs of the operated knee were undertaken as per a standard protocol: AP (anteroposterior) X-ray with the patient standing with both feet together and pointing towards the front such that the knee caps faced directly in front. All X-rays included a long length of tibia: at least ten centimeters below the tip of the prosthesis. The data was recorded on an electronic database (Microsoft Access).

The TKR were classified into 4 subgroups:

- standard instruments nonobese—nonobese STA,
- standard instruments obese—obese STA,
- computer assisted nonobese—nonobese CAS,
- computer assisted obese—obese CAS.

In the grouping process, the cutoff BMI considered was 30 kg/m^2^. Further subclassification as per the WHO grading into overweight, obese, and morbidly obese was not made.

Operative technique employed by the surgeon in all cases was similar. The tourniquet was inflated immediately prior to skin incision and released on completion of skin closure and pressure dressing. Tourniquet time was thus a fair measure of surgical time.

Tourniquet times of groups (A) and (B) and (C) and (D) were compared. The obese groups, that is, groups (B) and (D), were each divided into three equal subgroups, in chronological order. Mean tourniquet times of the corresponding subgroups within groups (B) and (D) were compared so as to obtain a chronological analysis of tourniquet time in obese patients.

There was a minimum five-year followup for all patients. Average return rate of the postal questionnaires was 89% over six years. 233 TKR completed the three-year clinical review. The available data for OKS, IKSS, and radiological alignment of the groups of TKR was compared. X-ray alignment of the tibial prostheses on AP X-ray was measured computing a mean of total four readings, that is, two different readings taken separately by two independent observers. Student *t*-tests were used for comparison of parameters between the groups with *P* value less than 0.05 taken as level of significance.

## 3. Results

**Figure 1:**
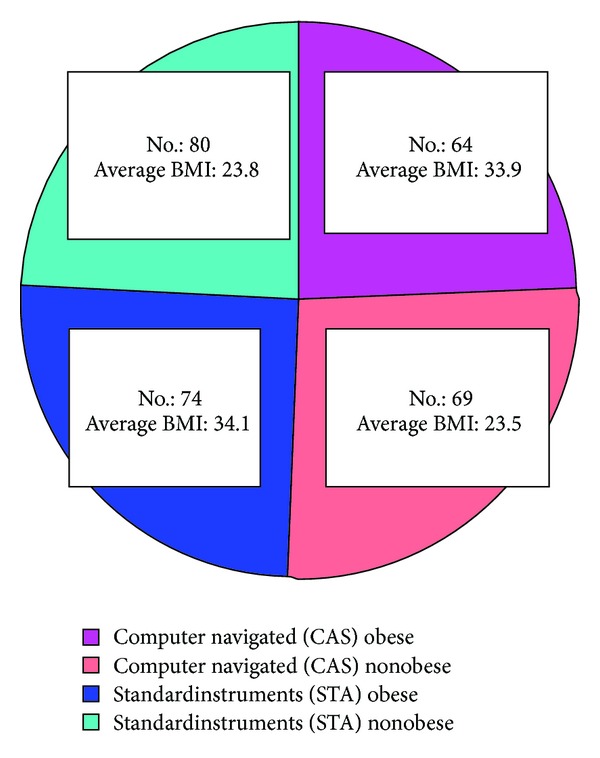
Distribution of TKR as per BMI (obesity) and operative technique.

Out of 287 TKR studied, 138 were found to be in the obese group and 149 in the nonobese group. Thus the prevalence of obese patients seeking TKR in our centre was 48%. 133 of the 287 TKR (44.6%) were performed with the assistance of computer navigation (CAS).  Figure 1 shows the distribution of TKR into the four groups with the number of TKR under consideration and average BMI of patients at surgery in each group, respectively. Sex distribution, average age, and average preop OKS of each group are shown in Table 1.

Table 2 shows average tourniquet times for surgery in all of our four groups. When we compared the tourniquet times of TKR performed with standard instruments in the obese and nonobese groups, we found the difference to be statistically significant. When the average tourniquet times of TKR undertaken with computer navigation in the obese and nonobese groups were compared, no statistically significant difference was revealed.

**Figure 2:**
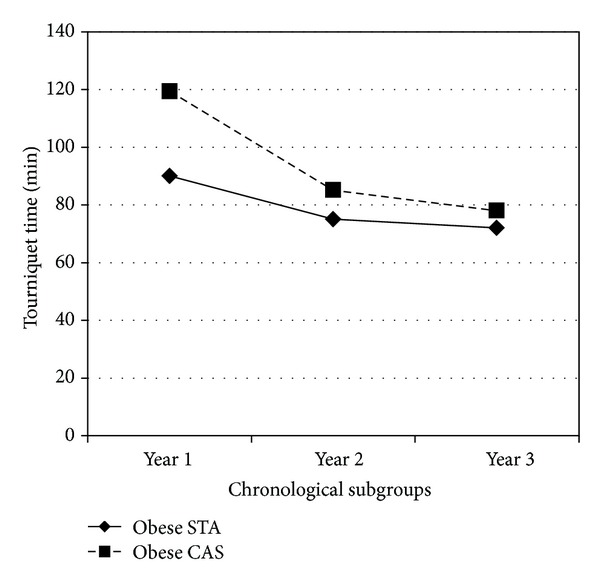
Tourniquet times of obese STA and obese CAS TKR over three chronological periods (designated as year as the periods were roughly a year each).

Figure 2 shows the analysis of the two obese groups that is, groups, (B) and (D) over time. When tourniquet times of the corresponding subgroups in chronological order were compared, we found a statistically significant difference in the first period (*P* = 0). In the second and third periods, the difference was not significant (*P* = 0.07 and 0.2, resp.); that is, with increasing experience, computer navigated TKR did not take a significantly longer time than TKR with standard instruments in obese patients.

**Figure 3:**
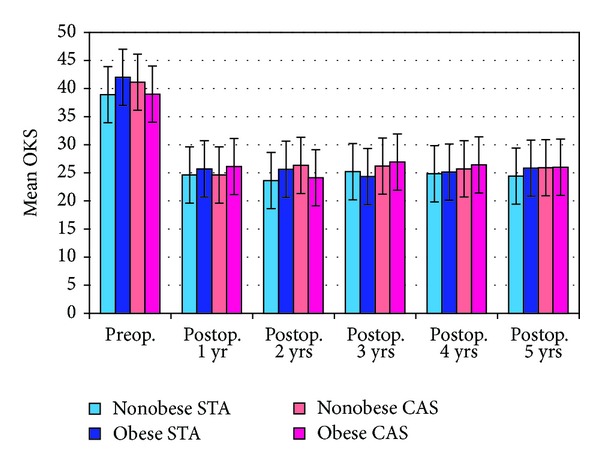
Mean OKS of the four groups of TKR over time.

Figure 3 shows the mean OKS of the four groups plotted over time. All groups showed a significant fall in the OKS from the pre- to the postoperative stages. However, there was no significant difference between the groups at any stage up to five years. Comparison of the IKSS scores at three years also did not reveal any significant difference between the groups (*P* = 0.32). Mean IKSS knee scores were all in the range of 84 to 88 and mean IKSS function scores were in the range of 74 to 80.

The X-ray data showed that the tibial prostheses were mal-aligned (i.e., more than three degrees from the mechanical axis) in a total of 24 out of 233 TKR. Of these, 20 had been performed using standard instruments and the remaining four were computer-navigated. Of the 20 mal-aligned standard instrumented TKR, 16 were found to be obese and the remaining four nonobese. Among the four mal-aligned computer navigated TKR, two were obese and two nonobese.

## 4. Discussion

Among patients seeking joint replacement surgery, the prevalence of obesity is much higher than that in the general adult population. It is reported to be 40–50% in various series [6, 16]. These figures are consistent with the prevalence of obesity in our group. Obesity has also been cited as being responsible for increasing the severity of arthritis and eventually the likelihood of total joint replacement surgery at a younger age [17, 18].

The high volume of work at our arthroplasty centre and a robust data gathering process allowed us to pick up a large sample size from a single knee surgeon's firm, with similar implants used in all cases. This ensured minimisation of confounding factors when comparison was made. Our outcome data correlates with that of recent studies [6, 19–21] that do not demonstrate any difference in outcome of joint replacement between obese and nonobese patients. In morbidly obese patients, significantly higher rates of focal osteolysis have been demonstrated [22] at more than 5 years postoperatively. Amin et al. [23], in a prospectively matched study, have shown inferior knee society scores and higher incidence of radiolucent lines in the morbidly obese group even in the early postoperative period.

Winiarsky et al. [24] have identified a number of possible technical problems faced during TKR surgery in morbidly obese patients. Assessment of limb alignment with standard jigs is more difficult in obese patients as the normal bony landmarks are obscured. There is also an identified risk of cutting the tibia in varus, caused by fat pushing extramedullary jig medially. One could argue that imageless navigation depends on the surgeon to mark a few bony pints as well. However navigation software computes prosthetic alignment with reference to a range of parameters thereby accounting for a cross-check mechanism. The software in our system (Galileo, Smith & Nephew Orthopaedics, UK) provided three numerical values of tibial rotational alignment with reference to (i) second metatarsal, (ii) tibial tuberosity, and (iii) orientation of the tibia relative to the femur. Dynamic mapping of hip range of motion marks the hip centre of rotation. The apex of the resulting cone is calculated with less than 1 mm error. Computer navigation assistance could hence save time that is wasted at every surgical step of making clinical judgment of alignment. Exposure and closure related difficulties would obviously not be altered.

With computer navigated TKR, it follows that the number of inaccurately aligned TKR would be less as observed with our results. More so where standard instruments had been used, the less-well-aligned TKR were mostly obese. In the computer navigation subgroups, the less accurately aligned TKR comprised obese and nonobese patients equally. We thus see a distinct advantage with computer navigation that has not been reported previously. In case of obese patients it provides a double-edged sword: better alignment that also comes without any time penalty.

## 5. Conclusions

- Obesity significantly increases the operating time for TKR undertaken with conventional instruments. However, in computer navigated TKR there is no significant difference in operating time between obese and nonobese patients.
- As the surgeon ascends up his learning curve with computer navigation, it may not take longer to perform TKR with navigation as compared to standard instruments in obese patients.

Thus computer navigation can help the surgeon to overcome jig alignment uncertainty without any time penalty.

## Figures and Tables

**Table 1:** Sex distribution and average age in groups.

Group	No. of males	No. of females	Av. age (yrs)	Av. preop OKS
Obese CAS	29	35	71.52	39.0
Obese STA	25	49	71.7	42.0
Nonobese CAS	30	39	73.6	41.1
Nonobese STA	36	44	72.47	38.9

CAS: computer assisted surgery, STA: standard instruments.

**Table 2:** Average tourniquet times.

Group	Av. tourniquet times (mins.)	Range	SD	*P* value
Obese STA	79.1	50–120	17.83	0.0004
Nonobese STA	66.9	40–120	18.02	

Obese CAS	94.2	60–143	17.48	0.09
Nonobese CAS	88.7	60–131	20.01	

CAS: computer assisted surgery, STA: standard instruments.
